# Opposing effects of mortality factors on progeny operational sex ratio may thwart adaptive manipulation of primary sex ratio

**DOI:** 10.1002/ece3.3071

**Published:** 2017-05-30

**Authors:** Gaétan Moreau, Eldon S. Eveleigh, Christopher J. Lucarotti, Benoit Morin, Dan T. Quiring

**Affiliations:** ^1^ Département de biologieUniversité de MonctonMonctonNBCanada; ^2^ Natural Resources CanadaCanadian Forest Service‐Atlantic Forestry CentreFrederictonNBCanada; ^3^ Population Ecology GroupFaculty of Forestry and Environmental ManagementUniversity of New BrunswickFrederictonNBCanada

**Keywords:** balsam fir sawfly, differential mortality, Hymenoptera, *Neodiprion abietis*, outbreak cycle stochasticity

## Abstract

Despite extensive research on mechanisms generating biases in sex ratios, the capacity of natural enemies to shift or further skew operational sex ratios following sex allocation and parental care remains largely unstudied in natural populations. Male cocoons of the sawfly *Neodiprion abietis* (Hymenoptera: Diprionidae) are consistently smaller than those of females, with very little overlap, and thus, we were able to use cocoon size to sex cocoons. We studied three consecutive cohorts of *N. abietis* in six forest stands to detect cocoon volume‐associated biases in the attack of predators, pathogens, and parasitoids and examine how the combined effect of natural enemies shapes the realized operational sex ratio. *Neodiprion abietis* mortality during the cocoon stage was sex‐biased, being 1.6 times greater for males than females. Greater net mortality in males occurred because male‐biased mortality caused by a pteromalid parasitic wasp and a baculovirus was greater and more skewed than female‐biased mortality caused by ichneumonid parasitic wasps. Variation in the susceptibility of each sex to each family of parasitoids was associated with differences in size and life histories of male and female hosts. A simulation based on the data indicated that shifts in the nature of differential mortality have different effects on the sex ratio and fitness of survivors. Because previous work has indicated that reduced host plant foliage quality induces female‐biased mortality in this species, bottom‐up and top‐down factors acting on populations can affect operational sex ratios in similar or opposite ways. Shifts in ecological conditions therefore have the potential to alter progeny fitness and produce extreme sex ratio skews, even in the absence of unbalanced sex allocation. This would limit the capacity of females to anticipate the operational sex ratio and reliably predict the reproductive success of each gender at sex allocation.

## 1 INTRODUCTION

Sex ratio at maturity, hereafter referred to as operational sex ratio (OSR), influences competition for mates and reproductive output (Darwin, 1871), as well as sexual selection under some circumstances (Klug, Heuschele, Jennions, & Kokko, 2010). By contrast with the primary sex ratio, the ratio at fertilization that is under direct control of natural selection (see reviews in Charnov, 1982; Hardy, 2002; Wrensch & Ebbert, 1993), OSR and the proximate causes of OSR bias after parental investment ended (i.e., juvenile, sex‐biased mortality) are poorly understood and have been the subject of few field studies. This is likely due to the belief that juvenile, sex‐biased mortality after parental investment ended has little impact on natural selection, a point of view that has recently been challenged (Shyu & Caswell, 2016).

In arrhenotokous hymenopterans, one of the groups for which variations in sex ratios have been extensively studied, unbalanced sex allocation at the progeny and population scales can occur because mated females produce a diploid female or a haploid male by opting to fertilize an egg or not (Flanders, 1942; White, 1954). Unmated females cannot select offspring sex and only produce haploid males. The primary sex ratio determined by sex allocation may then be distorted by sex‐biased mortality due to abiotic conditions (Su & Li, 1993; Wilkes, 1959), declines in food quality (Moreau, Quiring, Eveleigh, & Bauce, 2003; Morrill, Gabor, Weaver, Kushnak, & Irish, 2000; Wellings, Morton, & Hart, 1986), and interactions with conspecifics (Grbić, Ode, & Strand, 1992; Ode & Strand, 1995; Taylor, 1981). It is also assumed that sex‐biased mortality may be induced in hymenopterans by pathogens, predators, and parasitoids, but evidence has come from either (1) laboratory experiments (Kapranas, Wajnberg, & Luck, 2009; Lin & Ives, 2003; Salt, 1935); (2) field studies in which sex biases cannot be unambiguously attributed to the action of natural enemies because host population sex ratio prior to their action was undetermined (Bird, 1961; Carne, 1969; Griffiths, 1959; Houseweart & Kulman, 1976); or (3) manipulated field studies using a single type of enemy (i.e., predator, pathogen or parasitoid) (Holling, 1958; Yearian, Young, & Livingston, 1973), disregarding other enemies that may have negated or increased sex biases in natural conditions. Thus, the occurrence and impact of sex‐biased mortality due to multiple natural enemies in field populations of hymenopterans, and its impact on OSR, remain largely undetermined, although natural enemies are recognized as one of the most potent selective forces shaping animal life histories (Fritz & Simms, 1992; Johnson, 2001).

**Figure 1:**
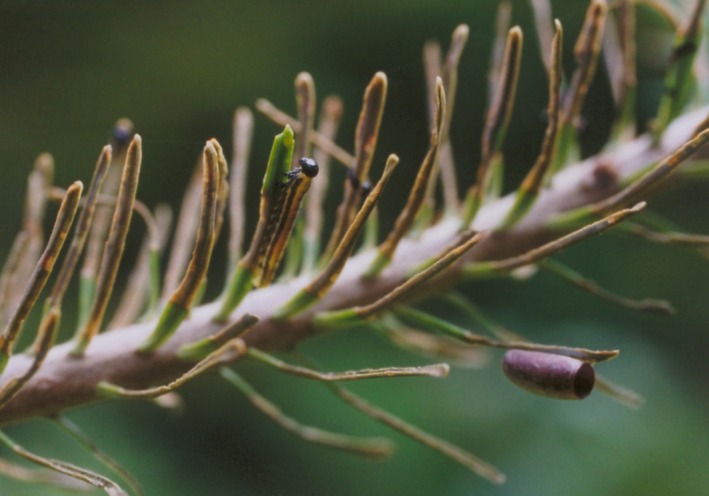
*Neodiprion abietis* larva, opened cocoon and foliage damage by *N. abietis* on a balsam fir (*Abies balsamea*) shoot

In ecological studies conducted with *Neodiprion abietis* (Harris) (Hymenoptera: Diprionidae) (Figure 1), a univoltine arrhenotokous sawfly with discrete generations (i.e., no parental care), OSRs ranged from 0.59 to 4.00 males per female (M:F) (Moreau, 2004). Although this wide range of OSRs could be attributed to unbalanced sex allocation, previous studies (Carroll, 1962; Moreau et al., 2003) have shown that cohorts collected from undefoliated host plants generally exhibited female‐biased sex ratios when successfully reared (i.e., low mortality) in the absence of natural enemies, suggesting that male‐biased sex ratios are probably due, at least in part, to female‐biased mortality in juveniles. Host–plant effects have been observed to induce sex‐biased mortality in *N. abietis*: reduced availability of different‐aged foliage (Moreau et al., 2003) and previous defoliation (Moreau & Quiring, 2011) induced greater mortality among females than males. Sex‐biased egg hatch due to deleterious mutations (Ebbert, 1993) or abiotic conditions has not been detected in *N. abietis* as hatching success is high and apparently only slightly reduced by parasitism (Huber & Moreau, 2003; Moreau, 2004). However, we suspected that the susceptibility of larvae and pupae of *N. abietis* to natural enemies may vary with sex because males and females of *N. abietis* differ in size and development time (Carroll, 1962; Moreau et al., 2003), as is commonly observed in Diprionidae (reviewed in Coppel & Benjamin, 1965); variations in both host size and development rate have been reported to influence host susceptibility to natural enemies in other systems (Fidgen, Eveleigh, & Quiring, 2000; Harvey & Strand, 2002; Williams, 1999).

Here, the specific questions that we address are as follows: (1) Can proximate factors that drive population fluctuations in *N. abietis* (Moreau, 2006), namely parasitoids, pathogens, and predators, cause sex‐biased mortality among juveniles in nature? (2) Knowing that host plant effects produce female‐biased mortality, can sex‐biased juvenile mortality be strong enough to explain severe OSR skews in natural sawfly populations? (3) Does differential mortality impact sawfly fitness? If answers to these questions are affirmative, sex‐biased mortality would appear to pose a serious constraint on the capacity of sawfly females to target an optimal OSR, despite their capacity to manipulate the primary sex ratio.

## 2 MATERIALS AND METHODS

In this study, we focus on the pupal/cocoon stage (Figure 1), the only juvenile stage that can be readily sexed in the field at its beginning and end due to sex‐biased differences in cocoon size (Carroll, 1962). Mortality occurring during the other developmental stages will be presented in a subsequent life‐table study (Moreau, G., Ostaff, D. P., Bauce, É., Eveleigh, E. S., Lucarotti, C. J., Morin, B., & Quiring, D. T., unpublished data). Most *N. abietis* larvae spin their cocoons directly on the foliage with no preference for a specific crown level or cardinal direction (Carroll, 1962). Removal of cocoons from foliage by predators, and cocoon dislodgement due to adult emergence, is infrequent in this species (Moreau, 2004). Thus, a sample of eclosed and uneclosed cocoons collected after adult emergence yields unbiased estimates of field survival during the cocoon stage, of sex ratio prior to and after the action of natural enemies, and of the distribution of cocoon volumes for each mortality factor.

Site conditions, sampling procedures, and determination of cause of mortality are similar to those described in Moreau et al. (2003), Moreau, Eveleigh, Lucarotti, and Quiring (2006a,b). We collected cocoons from 2000 to 2002 in six forest stands in western Newfoundland, Canada. In that period, this area was experiencing the most extensive *N. abietis* outbreak in the recorded history of outbreaks by this insect (Moreau, 2006). Stands were composed of naturally regenerated 25‐ to 35‐year‐old balsam fir (*Abies balsamea* (L.) Mill.) (over 90% of the basal area) growing at densities of 2,500 to 25,000 trees per hectare. Every year, at the end of adult emergence, we sampled one southwest‐facing branch from the mid‐crown of 25 balsam fir trees in each stand. We placed all eclosed and uneclosed cocoons present on the branches individually in gelatin capsules (Coni‐snap #00 natural; Wiler Fine Chemicals Ltd. London, Ontario). We measured cocoon length (θ ± 0.042 mm) and width (ω ± 0.042 mm) using a dissecting microscope fitted with a micrometer. Due to its oblong shape, we estimated cocoon volume from a calculation based on the volume of a cylinder (length = θ − ω; diameter = ω) terminated with a half sphere (diameter = ω) at both ends.

**Figure 2:**
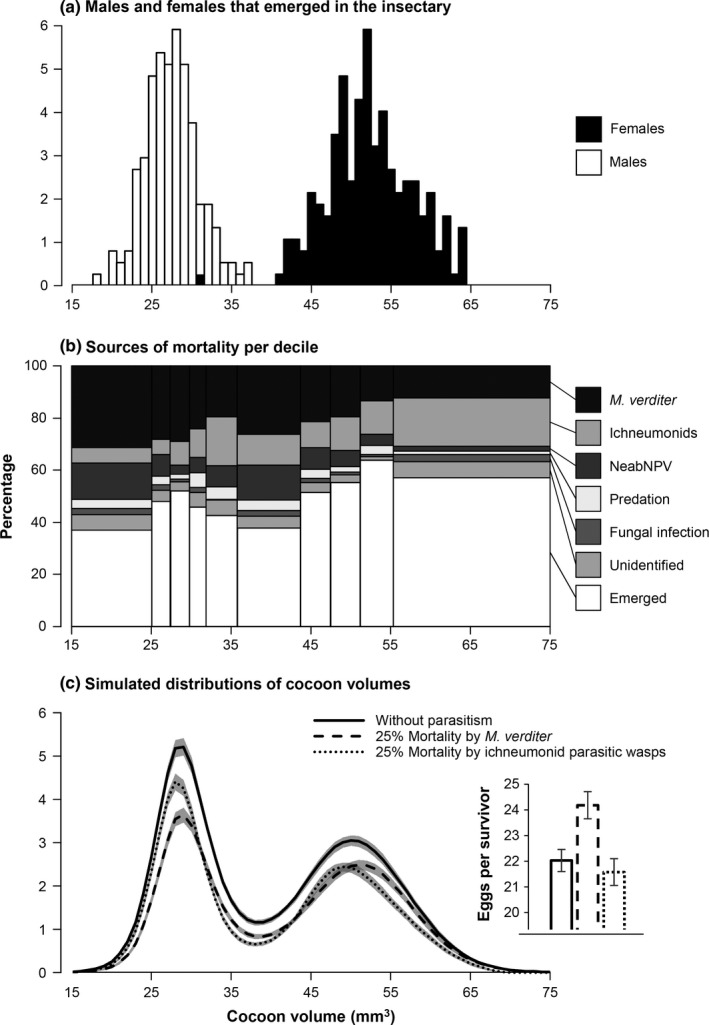
Distribution (%) of (a) cocoon volumes of *Neodiprion abietis* for males and females that emerged in the insectary from samples collected early in the cocoon stage (*n* = 889); (b) cocoon fates per decile for the whole field study (*n* = 3,142); (c) simulated cocoon volumes of *N. abietis* with a balanced sex ratio (1) in the absence of natural enemies, (2) that is then subjected to 25% mortality by the pteromalid parasitic wasp *Mesopolobus verditer* (dashed line), and (3) that is then subjected to 25% mortality by ichneumonid parasitic wasps (dotted line). In (c), areas highlighted in gray indicate the error associated with each GAM smoothing and the inset presents mean values (±SEM) of potential fecundity per survivor in simulated populations

To determine whether cocoon volume could be used to sex *N. abietis* pupae, using the same methodology, we collected a sample of 50 uneclosed cocoons early in the cocoon stage in each stand from 2000 to 2002 and reared them in gelatin capsules in an insectary. We sexed the adults following emergence and plotted the data to obtain the frequency distribution of cocoon volumes for males and females. The frequency distribution of cocoon volumes was bimodal (Figure 2a). The first mode (volumes <40 mm^3^) was largely comprised (≈99%) of males, and the second mode (volumes ≥ 40 mm^3^) was entirely comprised of females (Figure 2a). Consequently, we classified cocoons with volumes <40 mm^3^ as male and cocoons with volumes ≥ 40 mm^3^ as female.

We determined the incidences of predation and parasitism through visual inspection and dissection of cocoons. We classified *N. abietis* cocoons from which either an adult parasitoid had emerged or that contained remains of one or several parasitoid larvae or pupal case(s) as parasitized. If a parasitoid or parasitoid remains were located within the pupal case of another parasitoid (i.e., hyperparasitism), we attributed the mortality to the hyperparasitoid. Attributing pupal mortality to the hyperparasitoid and not the primary parasitoid had a negligible effect on results, as hyperparasitism occurred infrequently (see Section 3). We identified all adult parasitoids emerging in the laboratory from *N. abietis* cocoons by comparisons with voucher specimens from the Canadian National Collection (CNC), Ottawa. We compared cocoons from which adult parasitoids had emerged in the field prior to sampling to those containing parasitoid pupal cases and emergence holes made by adult parasitoids that had emerged in the laboratory to identify the parasitoids that had emerged in the field. We classified dead *N. abietis* within cocoons that were either ripped or had puncture holes with no sign of parasitism as dead from predation. We macerated in distilled water and homogenized the contents of uneclosed cocoons that showed no sign of parasitism or predation. We then examined a portion of the homogenate for entomopathogens at 400× magnification under a compound microscope and tested another portion for infection by the baculovirus NeabNPV using a molecular probe (see Moreau et al., 2005). Because NeabNPV‐induced mortality, followed by bacterial activity, often results in internal liquefaction of infected individuals (G. Moreau, personal observation), specimens that died from NeabNPV could not be dissected to determine if they were parasitized and therefore subjected to compensatory (i.e., overlapping) mortality. Similarly, we could not determine whether pupae killed by predators were parasitized or infected.

### 2.1 Data analysis

Unless otherwise mentioned, all analyses reporting a chi‐square value correspond to a 2 × 2 contingency table used to compare frequencies for cocoons with volumes <40 mm^3^ (i.e., males; see Section 3) and those with volumes ≥ 40 mm^3^ (i.e., females; see Section 3). We obtained all smoothed distributions of cocoon volumes using generalized additive models (GAMs) with the R package MGCV 1.8–6 (Wood, 2015). The dependent variable, the frequency of cocoons, was fitted using a Poisson distribution.

A preliminary GAM showed that for all sources of mortality, cocoon volume, years, and stands did not interact, indicating that cocoon volume frequencies affected by each natural enemy are undistinguishable among stands and years. Thus, data were pooled with respect to these two parameters. The percent mortality due to each natural enemy, however, varied between stands and years and will be discussed in a subsequent life‐table study (Moreau, G., Ostaff, D. P., Bauce, É., Eveleigh, E. S., Lucarotti, C. J., Morin, B., & Quiring, D. T., unpublished data). To support the GAM comparing the frequency distribution of cocoon volumes for the whole experiment with (1) each source of mortality and (2) cocoons from which adult *N. abietis* emerged, we divided the distribution of cocoon volumes into deciles (Figure 2b). Then, we used a two‐way frequency table to determine whether mortality/emergence was smaller or greater than what would be expected by chance for each volume decile, thus indicating differential mortality/survival with volume rank.

To determine how differential mortality by parasitism affects cocoon distribution and fitness, we simulated a cocoon distribution (*n* = 2,500; M:F ratio = 1:1). Then, we subjected this distribution to 25% mortality by Pteromalidae and by Ichneumonidae, which have been documented to parasitize up to 72 and 37% of the cocoons, respectively, in *N. abietis* field populations (Moreau, 2004). We generated the cocoon distribution by resampling the current dataset while excluding pupae dying from diseases, predation, or unknown factors. We obtained the potential fecundity of pupae with cocoon volumes ≥ 40 mm^3^ (i.e., females) by linear regression using a formula (oviposition = 59.05 × ln[cocoon volume] − 188.08; *r* ^2^ = .65; *F*_1,48_ = 90.49; *p* < .01) developed from oviposition data of a previous study (Moreau & Quiring, 2011).

## 3 RESULTS

A total of 3,142 *N. abietis* cocoons were recovered throughout the study. Mortality of *N. abietis* during the pupal stage was due to parasitoids, a baculovirus (i.e., NeabNPV), predation, and fungal infection (Table 1). Four percent of the pupae died from unidentified causes. Six different parasitoids were recovered from cocoons (Table 1). Hyperparasitism occurred <2% of the time and resulted from the pteromalid parasitic wasp, *Mesopolobus verditer*, attacking ichneumonid parasitic wasps within ichneumonid‐parasitized cocoons. Because of its important impact on *N. abietis* populations as a primary parasitoid, *M. verditer* will be dealt with separately from the five ichneumonid parasitic wasps in analyses.

**Table 1:** Sources of mortality affecting pupae during the study, and male‐to‐female sex ratio of affected pupae. All parasitoids recovered from cocoons were hymenopterans

Source	Species involved	M:F sex ratio of affected cocoons
Parasitism by Pteromalidae	*Mesopolobus verditer* (Norton)^a^	1.67:1^f^
Parasitism by Ichneumonidae	*Agrothereutes lophyri* (Norton)^b^	0.94:1^f^
	*Endasys patulus* (Viereck)^b^	
	*Itoplectis quadricingulata* (Provencher)^b^	
	*Gelis* sp.^b^	
	*Lamachus* sp.^b^	
Baculovirus	NeabNPV	1.62:1^f^
Predation	*Nabicula* sp. (Heteroptera: Nabidae)^c^	1.65:1
	Other species^d^	
Fungi	Several species^e^	1.50:1
Unknown		1.60:1

a Act as a primary parasitoid and as a hyperparasitoid. In concurrent work, *M. verditer* did not emerge from sawflies collected as larvae in the field and reared in the laboratory, strongly indicating that *M. verditer* only attacks cocoons.

b Act as a primary parasitoid.

c Responsible for most of the predation during the study (G. Moreau, personal field observation).

d Cocoons had a small hole that was probably caused by the stylet of a predator or by the ovipositor of a parasitoid.

e May have been pathogenic or saprophytic.

f Significantly differ from the sex ratio of the sum of all cocoons (i.e., 1.16:1 M:F; χ^2^ ≥ 3.83; *df* = 1; *p* ≤ .05).

**Figure 3:**
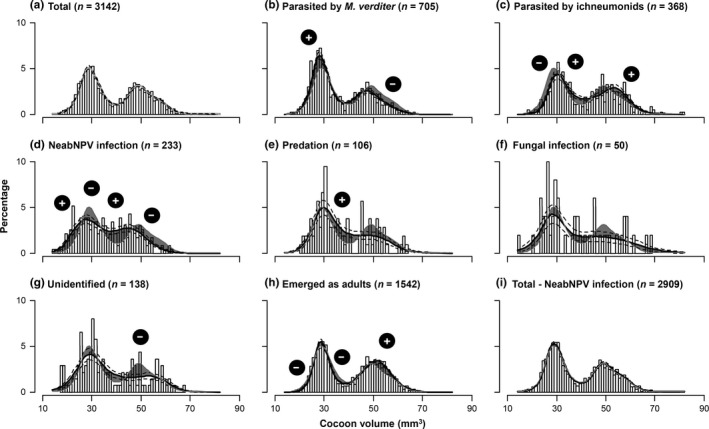
Frequency (%) distribution of cocoon volumes of *Neodiprion abietis*: (a) for the whole field study (total); (b) parasitized by the pteromalid parasitic wasp *Mesopolobus verditer*; (c) parasitized by ichneumonid parasitic wasps; (d) infected with NeabNPV; (e) killed by predators; (f) infected with fungal disease; (g) that died from unidentified causes; (h) cocoons from which adult *N. abietis* emerged; and (i) total distribution excluding NeabNPV‐infected individuals. The overlaid black lines illustrate the smoothed distribution of cocoon volumes, and dashed lines indicate the SEM. In (b–i), the difference between the overlaid smoothing and the smoothing in (a) is highlighted in gray. Frequencies that are significantly higher or lower (χ^2^ ≥ 4.09; *df* = 1; *p* ≤ .03) than would be expected by chance, as determined using a two‐way frequency table on deciles, are indicated by plus or minus symbols, respectively

Distributions of cocoon volumes are presented for all cocoons examined (Figure 3a), for cocoons associated with each source of mortality (Figure 3b–g), for cocoons from which *N. abietis* had emerged as adults (Figure 3h), and for cocoons per volume decile (Figure 2b). A GAM smoothing on the frequency of all cocoons (*r* ^2^ = .97; *edf* = 8.73; *df* = 8.98; χ^2^ = 1,394; *p* < .01) generated a bimodal distribution, with the two modes overlapping around a volume of 40 mm^3^ (Figure 3a), similar to the independent sample of small male and larger female cocoons discussed above (Figure 2a).

The predictions of a second GAM carried out per cocoon fate are presented in Figure 3b–g (*r* ^2^ = .95; *edf* ≥ 5.47; *df* ≥ 6.52; χ^2^ ≥ 38.04; *p* < .01). Male‐biased parasitism by *M. verditer* (Table 1) was the main source of pupal mortality (22.4%), occurring more frequently than would be expected by chance in small‐to‐intermediate‐sized male cocoons, and less frequently than would be expected by chance in large female cocoons (Figures 2b and 3b). Parasitism by the complex of five ichneumonids caused 11.7% mortality and was biased toward females (Table 1). In both modes, ichneumonids were more likely to attack larger cocoons, and in the mode mainly composed of males, ichneumonids rarely caused mortality in small‐to‐intermediate‐sized cocoons (Figures 2b and 3c). The baculovirus NeabNPV caused 7.4% mortality and was biased toward small cocoons; in both modes, NeabNPV mortality was greater than would be expected by chance in small individuals, and it was less frequent than would be expected in intermediate‐sized male and large female cocoons (Figures 2b and 3d). Mortality caused by predation, fungal infection, and other unknown factors occurred 9.4% of the time and was seldom associated with cocoon volume because frequencies were much lower (Figure 3e–g).

A comparison of the frequency distribution of all cocoons (Figure 3a) with that of the frequency distribution of cocoons from which sawfly adults had emerged (Figure 3h) indicates that pupal mortality was sex‐biased. Adult sawflies emerged from only 44% of cocoons <40 mm^3^ but from 55% of cocoons ≥40 mm^3^ (Figure 2b). Thus, the odds ratio of dying during the pupal stage was 1.6 times higher for males than females (χ^2^ = 37.78; *df* = 1; *p* < .01). This sex‐biased mortality caused the sex ratio to shift from male‐biased (1.16 M:F; Figure 3a) to female‐biased (0.93 M:F; Figure 3h) (significance of the shift: χ^2^ = 12.56; *df* = 1; *p* < .01). Higher survival rates of females were associated with lower mortality due to *M. verditer* and NeabNPV and higher mortality due to ichneumonids (Figure 2b). Conversely, lower survival rates of males were associated with higher mortality due to *M. verditer* and NeabNPV and lower mortality due to ichneumonids (Figure 2b). Greater net mortality in males occurred because male‐biased mortality caused by the pteromalid and the baculovirus was greater than female‐biased mortality caused by the complex of ichneumonids.

A comparison of the frequency distribution of laboratory‐reared (Figure 2a) and field‐collected (Figure 3a) cocoons revealed that a greater overlap occurred in the field between the distributions of male and female cocoon volumes. To determine whether NeabNPV could have caused the difference by reducing the size of some females below the 40 mm^3^ threshold, the total distribution was plotted again without the individuals killed by NeabNPV. The figure thus produced (Figure 3i) was nearly identical to the total distribution (Figure 3a), indicating that NeabNPV did not significantly bias the baseline distribution of cocoon volumes used in the analyses above.

We used a simulation with fixed parasitism rates to determine how the pteromalid *M. verditer* and ichneumonids can shape cocoon distributions when the initial sex ratio is 1:1 M:F and no other mortality factor is involved. The simulation shows that a parasitism rate of 25% by *M. verditer* reduced the frequencies of male and small female cocoons, causing the OSR to shift from 1:1 to 0.84:1 M:F (χ^2^ = 7.33; *df* = 1; *p* < .01) (Figure 2c). By contrast, a parasitism rate of 25% by ichneumonids reduced the frequencies of female and large male cocoons but was not as skewed as with *M. verditer*, causing an insignificant shift in OSR from 1:1 to 1.02:1 M:F (*p* = .74) (Figure 2c). A GAM indicated that cocoon frequencies following parasitism by *M. verditer* or ichneumonids differed in terms of intercept (χ^2^ = 21.73; *df* = 1; *p* < .01) and distribution (χ^2^ = 33.60; *df* = 2; *p* < .01). Male‐biased mortality caused by the pteromalid caused the potential fecundity per survivor to increase by 10% (χ^2^ = 8.95; *df* = 1; *p* < .01), while female‐biased mortality caused by ichneumonids insignificantly reduced potential fecundity per survivor by 2% (*p* = .51) (Figure 2c).

## 4 DISCUSSION

Natural populations of *N. abietis* were subjected to sex‐biased mortality due to natural enemies, with different natural enemies affecting host population OSRs in different directions (i.e., both male‐biased and female‐biased) in a single life stage. Little is known about sex‐biased mortality in most nonhuman species (Carey & Liedo, 1995), and to the best of our knowledge, only one study on a lepidopteran (e.g., Fuester & Taylor, 1996) has reported sex‐biased impacts of natural enemies in natural populations of juveniles. We believe that this is the first study to report such results in a species that can directly manipulate the primary sex ratio of offspring in response to environmental pressure. This is of particular significance when considering that reduced host plant foliage quality, by itself, induced female‐biased mortality during larval stages in this species that were shown to shift OSRs from female‐biased (i.e., 0.8:1 M:F) to male‐biased (i.e., 3.3:1 M:F) (Moreau et al., 2003). Thus, bottom‐up (host plant) and top‐down (natural enemies) factors acting on *N. abietis* can affect OSRs in similar or opposite directions. Shifts in ecological conditions therefore have the potential to produce the wide range of sex ratios observed in natural populations of this species, even in the absence of unbalanced sex allocation.

Trivers and Willard (1973) originally suggested that parents should adjust the primary sex ratio of their offspring in response to environmental conditions. For this to be possible, however, parents must be able to predict the reproductive success of each gender and have control over the primary sex ratio (Trivers & Willard, 1973). Arrhenotokous *N. abietis* females meet these criteria to some extent because they can manipulate offspring sex ratio, if mated, and have been observed to preferentially oviposit on host plants exhibiting levels of defoliation that reduce sex‐biased mortality (G. Moreau, personal observation). However, at the time of oviposition, females possess little information about the prevalence of natural enemies that will affect juvenile sex ratios and fitness, 10–11 months later, because *N. abietis* juvenile mortality rates are known to fluctuate tremendously from one year to the next (Moreau et al., 2006a,b). We have shown that pupal mortality is a major determinant of sex ratio and fecundity, which could limit the capacity of females to anticipate the OSR and reliably predict the fitness of each gender. We thus speculate that the unpredictable nature and strong impact of the mortality associated with natural enemies directly contribute to the large departure from a 1:1 M:F OSR that is associated with this species.

### 4.1 Sources of differential mortality

As with several other Diprionidae (Lyons & Griffiths, 1962), *N. abietis* males pupate a week earlier than females but both sexes emerge synchronously from cocoons (Carroll, 1962). As a result, females have a longer larval development period than males and males have a longer pupal development period than females. Because *M. verditer* attacks cocoons, whereas most ichneumonids attack larvae (Table 1; Carroll, 1962), male‐biased mortality by *M. verditer* and female‐biased mortality by ichneumonids may be associated with differences in the exposure time of each sex to the parasitoids. A second explanation for biases in parasitoid mortality is that ichneumonid parasitoids and *M. verditer* may prefer larger and smaller hosts, respectively, as was previously documented in laboratory studies with hymenopteran parasitoids (Lin & Ives, 2003). A third explanation for biases in parasitoid mortality is that ichneumonid selection of larger larval hosts reduced the availability of larger cocoon hosts for *M. verditer*, causing the latter to select smaller cocoons. However, this last explanation is unlikely to explain our results because previous parasitism by ichneumonids had little effect on the availability of large unparasitized female hosts (Figure 3h) and because *M. verditer* can readily attack cocoons containing parasitoids (i.e., hyperparasitism) or hyperparasitoids (Eveleigh et al., 2007). Therefore, variations in the susceptibility of each sex to each type of parasitoid are best explained by differences in size and life histories of male versus female hosts.

The absence of obvious modes in the distribution of cocoon volumes for individuals killed by NeabNPV and fungal infection suggests that the apparent bias in the mortality attributable to these factors may also be due, at least in part, to a decline in the size of infected individuals, as was observed in other systems (Myers, Malakar, & Cory, 2000; Rothman & Myers, 1996). Female‐biased (Santiago‐Alvarez & Vargas‐Osuna, 1986; Tsuey & Ma, 1993; Yearian et al., 1973) juvenile mortality by nucleopolyhedroviruses (NPVs) has been previously reported in several other systems and has generally been attributed to differences in the duration of the developmental period. To our knowledge, male‐biased juvenile mortality by NPVs has not been documented.

### 4.2 Consequences of differential mortality for population dynamics

Although this study only focused on one developmental stage of *N. abietis*, the findings have implications for the population dynamics of the species as a whole. First, they indicate that proximate factors driving population fluctuations in *N. abietis* (Moreau, 2006) can cause sex‐biased mortality among juveniles in nature. Because these mortality factors have been shown to vary in effect during a *N. abietis* outbreak (Moreau & Quiring, 2011; Moreau et al., 2006a), they could modulate the OSR. This would offer a mechanism to explain a previous observation (Carroll, 1962) that *N. abietis* sex ratio apparently changes with the outbreak stage.

It is well known that preference of natural enemies for larger hosts may increase the population growth rate of natural enemies (Charnov, 1982; Luck, Stouthamer, & Nunney, 1993). This field study suggests that sex biases in the attack of natural enemies may also affect host population growth rate through its impact on host OSR and fecundity. And although arrhenotoky in *N. abietis* may permit unmated females to produce offspring when male mates are scarce or absent, unmated females produce only male progeny, which could result in further biases in OSRs. Ultimately, these effects can indirectly contribute to the relative stochasticity of *N. abietis* outbreaks (Moreau, 2006). Whether differential mortality plays a role in outbreak cycle stochasticity of other outbreaking sawfly species (Larsson, Björkman, & Kidd, 1993) remains to be tested.

### 4.3 Final considerations

According to Fisher (1930) and several others, juvenile sex‐biased mortality after parental investment ended should not affect the primary sex ratio. By contrast, Shyu and Caswell (2016) have recently suggested that sex‐biased mortality should cause the primary sex ratio to be biased toward the lower mortality sex. This implies that sex‐biased mortality is somewhat predictable and leads to known OSR. However, based on our work, we suggest that sex‐biased mortality can be too unpredictable for selection to aim for a given OSR.

## DATA ACCESSIBILITY

Data are available on DRYAD (https://doi.org/10.5061/dryad.4c058).

## CONFLICT OF INTEREST

None declared.
